# The influence of plate removal on functional and patient reported outcomes after scaphoid nonunion surgery

**DOI:** 10.1177/17531934251413137

**Published:** 2026-01-15

**Authors:** Philip M J Schormans, Anna R Y Van der Heijden, Martijn Poeze, Jan A Ten Bosch, Pascal F W Hannemann

**Affiliations:** 1 Department of Surgery, Amphia Hospital, The Netherlands; 2 Department of Hand and Wrist Surgery, Maastricht University Medical Center, The Netherlands

**Keywords:** Functional outcomes, plate fixation, plate removal, scaphoid non-union

## Abstract

**Introduction:**

Volar locking plate fixation with cancellous bone grafting is an effective treatment for scaphoid non-union. However, the plate can impinge on the volar rim of the radius, causing pain or restricted movement that often necessitates plate removal. The primary aim of this study was to evaluate the effect of hardware removal on wrist function and patient-reported outcomes after plate fixation for scaphoid non-union.

**Methods:**

This prospective study assessed 49 of 113 patients who underwent plate fixation for scaphoid non-union and later required plate removal due to functional impairment. Range of flexion and extension, grip strength and Patient-Rated Wrist and Hand Evaluation (PRWHE) scores were measured before non-union surgery, after bone union but before plate removal, and at 3 months after plate removal.

**Results:**

A decrease in wrist flexion after the initial non-union surgery (60–48°) was reversed by plate removal (48–65°). Extension and grip strength increased significantly compared with pre-operative values (54–65° and 66–88%). Patient-reported outcomes also showed marked improvement, with PRWHE scores improving from 36 preoperatively to 23 after union, and finally to 4 after plate removal. No complications related to plate removal were observed.

**Conclusion:**

Plate removal in patients with functional impairment after scaphoid non-union surgery produces clinically meaningful improvements in wrist movement and patient-reported outcomes.

**Level of evidence:**

Level III

## Introduction

Undisplaced scaphoid waist fractures usually unite with non-operative management, but non-union remains a common complication (Dy et al., 2018). The optimal treatment of scaphoid non-union has been extensively debated for decades, and many surgical techniques have been described. We have previously described the use of volar plate fixation with autologous cancellous bone grafting in two prospective cohort studies of 21 and 49 patients, respectively, (Schormans et al., 2018; Schormans et al., 2020). These studies showed good midterm outcomes in radiological union, range of motion, grip strength and patient-reported outcome measures (PROMs). Despite these outcomes, a significant proportion of patients (37%) underwent plate removal owing to perceived functional limitations or impingement against the volar lip of the distal radius (Schormans et al., 2020).

Plate removal surgery in other series of volar plate fixation for scaphoid non-union varies between 20 and 65% (Dodds et al., 2018; Mehling et al., 2019). The most common reasons for plate removal were symptomatic functional deficit or impingement pain.

Routine removal of implants after uneventful fracture healing remains controversial. There are currently no guidelines or evidence-based recommendations specific to routine implant removal after plate fixation of scaphoid non-unions (Yuan et al., 2022). As volar plate fixation of the scaphoid has become increasingly popular in recent years, it is important to determine whether plate removal provides measurable improvement in wrist range of motion and PROMs (Mehling et al., 2019).

The primary aim of this prospective cohort study was to evaluate changes in range of motion and patient-reported outcomes after volar plate removal in patient cohort previously treated for scaphoid non-union using volar miniplate fixation and cancellous bone grafting. We hypothesized that removal of the volar plate in patients with healed scaphoid non-union and persistent functional impairment would result in significant improvements in range of motion and patient-reported outcomes at 3 months after hardware removal compared with both pre-plate fixation and pre-removal values.

## Methods

This study was registered and approved by the institution’s medical ethical committee and was conducted according to the Declaration of Helsinki (World Medical Association, 2013).

**Figure 1.**
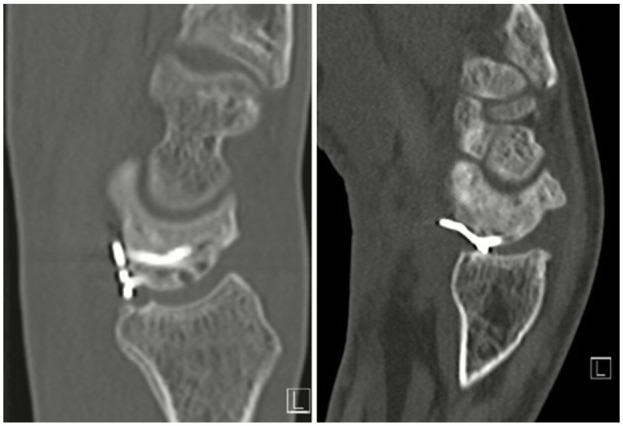
Impaction of the plate against the volar rim of the distal radius (left) and interposition of the plate in the radiocarpal joint (right).

Between November 2013 and August 2024, 113 patients who presented to our tertiary referral centre with a scaphoid non-union were enrolled in a prospective cohort study of volar angular-stable plate fixation. This study included all patients from this cohort who underwent plate removal surgery after union of the scaphoid. Inclusion criteria for plate removal after scaphoid non-union surgery were a subjective functional deficit, persistent pain owing to impingement of the plate on the volar rim of the distal radius or radiological evidence of plate impaction against the volar radius (Figure 1). Patients unwilling to undergo plate removal surgery were excluded.

Initial non-union surgery was performed using a 1.5 mm precontoured variable-angle locking miniplate (Medartis AG, Basel, Switzerland) with the addition of cancellous bone graft from the ipsilateral iliac crest after debridement of the non-union and correction of the humpback deformity as previously described (Schormans et al., 2018, 2020). Plate removal was performed via the same volar modified Henry approach as used for the initial surgery (Conti Mica et al., 2017).

Function and patient-reported outcomes were assessed before the initial non-union surgery, after bony union on the day of plate removal and at three months after plate removal. Range of movement was measured using a handheld goniometer and expressed as the total degrees of active extension and flexion. Because of the variable effect of scaphoid non-union on ulnar and radial deviation, these measurements were not included in our study (Gehrmann et al., 2016). Grip strength was assessed using a Jamar dynamometer (Sammons Preston Rolyan, Illinois) and expressed as a percentage of the contralateral side. The Patient Rated Wrist and Hand Evaluation (PRWHE) questionnaire was used to determine patient-reported outcomes. The PRWHE is a 15-item questionnaire assessing patient perceived pain and function (Hoang-Kim et al., 2011; MacDermid and Tottenham, 2004).

Radiological scaphoid union was confirmed on multiplanar reconstructed computed tomography (Sanders, 1988), with criteria of at least 25% trabecular bridging after 12–18 weeks (Singh et al., 2005). Union was determined by consensus between an orthopaedic trauma surgeon with Level 5 expertise (PH) and an experienced musculoskeletal radiologist (DL), independently.

Any deviation from the normal postoperative course without need for additional surgical or pharmacological intervention was deemed a minor event. All other complications were regarded as major events.

### Statistical analysis

Data were imputed using multiple imputation for outcomes where data were missing. Missing values were below 30% for all variables. For questionnaire domains, missing values were imputed based on baseline characteristics and existing PRWHE items. For each missing value five imputations according to the Bayesian probability rules were performed. For the analysis of non-parametric parameters, the Wilcoxon signed rank test was used. Significance was set at *p* < 0.05. For all parameters, means and SD were calculated and reported.

## Results

The characteristics of the patients are summarized in Table 1. Compared with our previously published studies, patients with persisting functional impairments were more likely to have a proximal pole fracture (29 and 39% respectively) (Schormans et al., 2018, 2020).

**Table 1.** Characteristics of included patients.

Characteristic	Total (*n* = 49)
Mean age, years (range)	25 (15–60)
Male sex, *n*	43
Active smoker, *n*	16
Mean duration of non-union, months (range)	32 (4–192)
Sclerotic non-union, *n* (%)	29
Proximal pole non-union, *n* (%)	19
Humpback deformity, *n* (%)	28
DISI, *n* (%)	17
Stage I SNAC, *n* (%)	14
Previous surgery to scaphoid, *n* (%)	8
Mean time until union after volar plate osteosynthesis, months (range)	5.5 (3–22)

DISI: dorsal intercalated segment instability; SNAC: scaphoid non-union advanced collapse.

**Figure 2.**
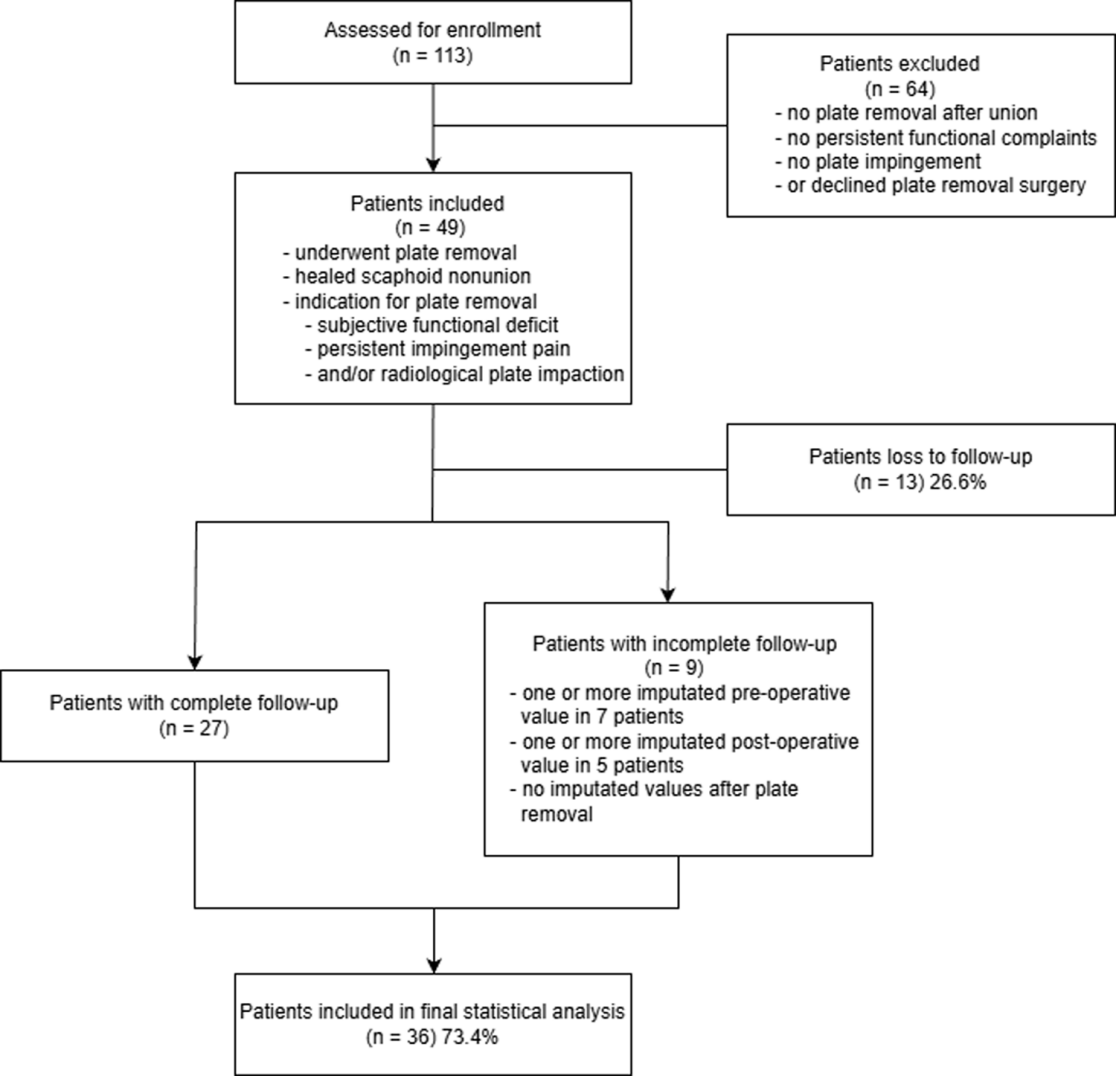
Flow diagram illustrating patient inclusion, exclusion, follow-up and handling of missing data in a prospective cohort study evaluating volar plate removal after scaphoid non-union surgery.

Out of 113 patients that underwent volar plate fixation for scaphoid non-union, 49 patients (43%) underwent plate removal. Of these, 36 patients (73.4%) were available for long-term follow-up at a mean of 61 months after non-union surgery (range 12–118 months) (Figure 2).

Functional and patient reported outcomes are summarized in Table 2. In patients with persisting pain or functional deficits after non-union surgery, active range of motion, and in particular active wrist flexion, decreased significantly (60–48°; *p* = 0.019).

**Table 2.** Functional and patient reported outcomes before and after plate removal.

Parameter	Preoperative, mean (SD)	Before plate removal, mean (SD)	After plate removal, mean (SD)	*p*-Value before and after plate removal (SD)*
Range of motion (deg)	115 (38)	100 (26)	128 (31)	<0.001 (<0.001)
Flexion (deg)	60 (23)	48(18)	65 (15)	<0.001 (<0.001)
Extension (deg)	54 (21)	54 (14)	65 (16)	0.006 (0.002)
Grip strength %	66 (19)	74 (16)	88 (16)	0.041 (0.021)
PRWHE pain score	22 (11)	14 (10)	3 (4)	0.008 (0.003)
PRWHE function score	27 (21)	18 (22)	1 (1)	0.008 (0.043)
PRWHE total score	36 (20)	23 (21)	4 (4)	0.005 (0.011)

PRWHE: Patient-Rated Wrist and Hand Evaluation; SD: standard deviation.

* Wilcoxon signed rank test (paired samples *t*-test, two-sided *p*).

After plate removal, statistically significant improvements were observed in active flexion (48–65°; *p* < 0.001), extension (54–65°; *p* = 0.006) and grip strength (74–88%; *p* = 0.041). When considering final functional outcome, all functional outcome parameters improved, although only extension and grip strength showed statistically significant improvement (54–65°, *p* = 0.006 and 66–88%, *p* < 0.001, respectively).

Patient reported outcomes improved significantly. Mean total PRWHE score improved after non-union surgery (36–23 points, *p* = 0.028). Additional statistically significant improvement in mean PRWHE score was seen after plate removal (23–4 points, *p* = 0.005). Overall improvements in PRWHE were statistically significant (36–4 points, *p* = 0.008) as well as clinically relevant, since the differences in PRWHE outcomes were beyond the minimal clinical important difference for scaphoid non-union surgery (Hoogendam et al., 2022).

No complication related to plate removal occurred.

## Discussion

Three months after hardware removal, flexion, extension and grip strength improved significantly in patients with persisting functional deficits after plate fixation of scaphoid non-union. Furthermore, a clinically relevant improvement in patient reported outcomes was observed in our cohort 3 months after plate removal. Our group previously published two prospective series of patients treated for scaphoid non-union using the same plate, and reported promising radiological, functional and patient-reported outcomes (Schormans et al., 2018, 2020). Other authors have observed similar results, with union rates between 87 and 100% and satisfactory functional and patient-reported outcomes (Dodds et al., 2014; Esteban-Feliu et al., 2018; Ghoneim, 2011; Putnam et al., 2019; Shin et al., 2024; Welle et al., 2022; Wu et al., 2019). The relatively high union rates, especially in patients with recalcitrant or secondary non-unions, can be attributed to high interfragmentary stability and preservation of a maximum surface area for bone healing ((Goodwin et al., 2019) Hart et al., 2013; Jurkowitsch et al., 2016).

However, a disadvantage of volar plate fixation for scaphoid non-unions is the possibility of impingement against the volar lip of the distal radius, which may cause functional limitations, pain and discomfort (Goodwin et al., 2018; Lemke et al., 2023; Shanbhag et al., 2021).

A systematic review on scaphoid plating for comminuted fractures found a mean plate removal rate of 21%, with hardware related symptoms cited as the reason for removal in the majority of studies (Morgan et al., 2021). Others have reported a removal rate of up to 65% owing to impaction of the plate against the distal radius or protruding screws (Lemke et al., 2023; Mehling et al., 2019). In this study, 43% of all plates were removed, consistent with these previous reports.

Marzella et al. (2024) reported a retrospective series of 44 patients treated with anterior locked plating, with plate removal in all cases regardless of symptoms or range of motion. They observed a significant improvement in range of motion, particularly wrist flexion. In our cohort, flexion and extension both improved after plate removal. The loss of flexion seen after initial plate fixation was reversed with plate removal. Plate fixation does not greatly affect active extension, but the final increase in extension after plate removal leads to significant improvement in overall extension.

Grip strength improved after plate removal, despite minimal change after the initial non-union surgery. Patient-reported outcomes also improved significantly after plate removal in this cohort. Although there was already a notable improvement in PRWHE after fixation of the scaphoid non-union, this improved further after plate removal. The overall change was greater than the minimal clinically important difference for scaphoid non-union surgery (Hoogendam et al., 2022), supporting a meaningful improvement from the patient’s perspective.

In our cohort, plate removal after successful scaphoid non-union surgery was associated with a statistically and clinically relevant improvement in both functional and patient-reported outcomes. However, as our study included only patients who underwent plate removal, we cannot draw conclusions regarding those who retained the implant. While routine removal cannot be universally recommended, elective plate removal should be considered in patients who experience residual symptoms or functional limitation after healing. One other study reported similar improvements in movement (Marzella et al., 2024), although this cohort included only patients with acute scaphoid fractures, and therefore did not include baseline functional or PROM data. The independent contribution of plate removal in this series remains unclear.

This study is limited by the nonresponse rate of 27% at long-term evaluation, potentially introducing selection bias. In addition, the study includes only patients who underwent plate removal; outcomes of those who retained the implant remain unknown, which limits the generalizability of our findings. Despite a mean follow-up of 61 months, no conclusions can be drawn regarding the effectiveness of this surgical technique in preventing long-term complications of scaphoid non-union. Finally, comparing our results with other techniques remains challenging owing to heterogeneity in inclusion criteria, operative and perioperative policies, and reported outcomes.

Our statistical analysis is limited by the use of multiple pairwise tests across three time points rather than a repeated-measures model, increasing the risk of type 1 error. The small sample size and proportion of missing data constrained the robustness of the multiple imputation model, including the limited number of imputations and uncertainty regarding missing-data assumptions. Because non-parametric methods were applied, pooling across imputed datasets and sensitivity analyses were not feasible, which may reduce the precision of the estimated effects.

In conclusion, plate removal can meaningfully improve wrist movement and patient-reported outcomes in patients with plate-related symptoms or radiological impingement after scaphoid non-union surgery.
